# Novel Aza-podophyllotoxin derivative induces oxidative phosphorylation and cell death via AMPK activation in triple-negative breast cancer

**DOI:** 10.1038/s41416-020-01137-4

**Published:** 2020-11-03

**Authors:** Dhanir Tailor, Catherine C. Going, Angel Resendez, Vineet Kumar, Dhanya K. Nambiar, Yang Li, Arpit Dheeraj, Edward Lewis LaGory, Ali Ghoochani, Alisha M. Birk, Tanya Stoyanova, Jiangbin Ye, Amato J. Giaccia, Quynh-Thu Le, Rana P. Singh, George W. Sledge, Sharon J. Pitteri, Sanjay V. Malhotra

**Affiliations:** 1 Department of Radiation Oncology, Stanford University School of Medicine, Palo Alto, CA 94304 USA; 2 Department of Cell, Development and Cancer Biology, Oregon Health & Science University, Portland, OR 97201 USA; 3 Center for Experimental Therapeutics, Knight Cancer Institute, Oregon Health & Science University, Portland, OR 97201 USA; 4 Department of Radiology, Canary Center at Stanford for Cancer Early Detection, Stanford University School of Medicine, Palo Alto, CA 94304 USA; 5 School of Life Sciences, Jawaharlal Nehru University, New Delhi, 110067 India; 6 Department of Medicine, Stanford University School of Medicine, Palo Alto, CA 94304 USA; 7 Stanford Cancer Institute, Stanford University School of Medicine, Stanford, CA 94305 USA

**Keywords:** Cancer, Breast cancer

## Abstract

**Background:**

To circumvent Warburg effect, several clinical trials for different cancers are utilising a combinatorial approach using metabolic reprogramming and chemotherapeutic agents including metformin. The majority of these metabolic interventions work via indirectly activating AMP-activated protein kinase (AMPK) to alter cellular metabolism in favour of oxidative phosphorylation over aerobic glycolysis. The effect of these drugs is dependent on glycaemic and insulin conditions.  Therefore, development of small molecules, which can activate AMPK, irrespective of the energy state, may be a better approach for triple-negative breast cancer (TNBC) treatment.

**Methods:**

Therapeutic effect of SU212 on TNBC cells was examined using in vitro and in vivo models.

**Results:**

We developed and characterised the efficacy of novel AMPK activator (SU212) that selectively induces oxidative phosphorylation and decreases glycolysis in TNBC cells, while not affecting these pathways in normal cells.   SU212 accomplished this metabolic reprogramming by activating AMPK independent of energy stress and irrespective of the glycaemic/insulin state. This leads to mitotic phase arrest and apoptosis in TNBC cells. In vivo, SU212 inhibits tumour growth, cancer progression and metastasis.

**Conclusions:**

SU212 directly activates AMPK in TNBC cells, but does not hamper glucose metabolism in normal cells. Our study provides compelling preclinical data for further development of SU212 for the treatment of TNBC.

## Background

Triple-negative breast cancer (TNBC) is the most aggressive form of breast cancer and accounts for 15–20% of total diagnosed breast cancer cases with high rates of metastasis and recurrence.^1^ Glucose metabolism and its reprogramming are key features of cancer cells. The increased proliferation exhibited by cancer cells is accompanied by increased demands for energy and anabolic substrates. Many cancers adapt to these demands via a metabolic shift known as the Warburg effect, in which oxidative phosphorylation is suppressed in favour of aerobic fermentation of glucose to lactate.^2^ AMPK activation antagonises the Warburg effect via repression of hypoxia-inducible factor 1α (HIF-1α) and the associated glycolytic effectors.^3^ This metabolic alteration is also associated with poor prognosis and the development of treatment resistance.^3^ In addition, the end product of aerobic glycolysis is lactate that plays an important role in the development of the treatment-resistant tumour microenvironment.

AMPK is a highly conserved energy sensor found in all eukaryotes.^4^ The AMPK pathway integrates diverse energetic inputs with a myriad of regulatory outputs that coordinate reduction in glycolysis, inhibit gluconeogenesis, cause cell-cycle arrest, decrease protein synthesis and increase mitochondrial biogenesis to correct energy deficits.^3,4^ AMPK activation also inhibits the enzymes associated with fatty acid and cholesterol synthesis, including acetyl-CoA carboxylase 1 (ACC1) and HMG-CoA reductase (HMGCR). Studies have shown that cancer cells undergo endogenous lipogenesis to improve their survival and drug resistance.^5^ AMPK is a heterotrimeric complex consisting of α, β and γ subunits, in which α is catalytic and β and γ are regulatory subunits. AMPK is activated by phosphorylation at Thr172 of the α subunit. This phosphorylation is regulated in part by liver kinase beta 1 (LKB1), but found altered in many cancer cases. AMPK suppresses the mammalian target of rapamycin complex 1 (mTORC1), which leads to decreased proliferation.^6^ mTORC1 is associated with the synthesis of macromolecules that are required for cell proliferation. AMPK activators, including metformin, predominantly activate AMPK through calorie restriction, and their effects are also dependent on the cell’s glycaemic and insulin state.^7^ Furthermore, metformin’s therapeutic effects on breast cancer metabolism and viability are not only highly dose-dependent but also saturable by physiologically relevant high concentrations of glucose and insulin, mimicking hyperglycaemia and hyperinsulinaemia, respectively.^7^ To overcome this limitation, energy stress-independent AMPK activators, including A-769662 (Abbott Laboratories), have been reported to activate AMPK independent of glycaemic or insulin state. Although these compounds activate AMPK, they are toxic on normal cells and show differential effects in cancer cells, presenting a need for new AMPK activators with better tolerability.^7^

Herein, we report the potential of a novel podophyllotoxin derivative to directly activate AMPK with selective anticancer toxicity while also modulating the Warburg effect in TNBC cells in vitro and in vivo in a manner that increases apoptosis and decreases tumour burden.

## Methods

### Synthesis of Aza-podophyllotoxin derivative and drug preparation

**Fig. 1:**
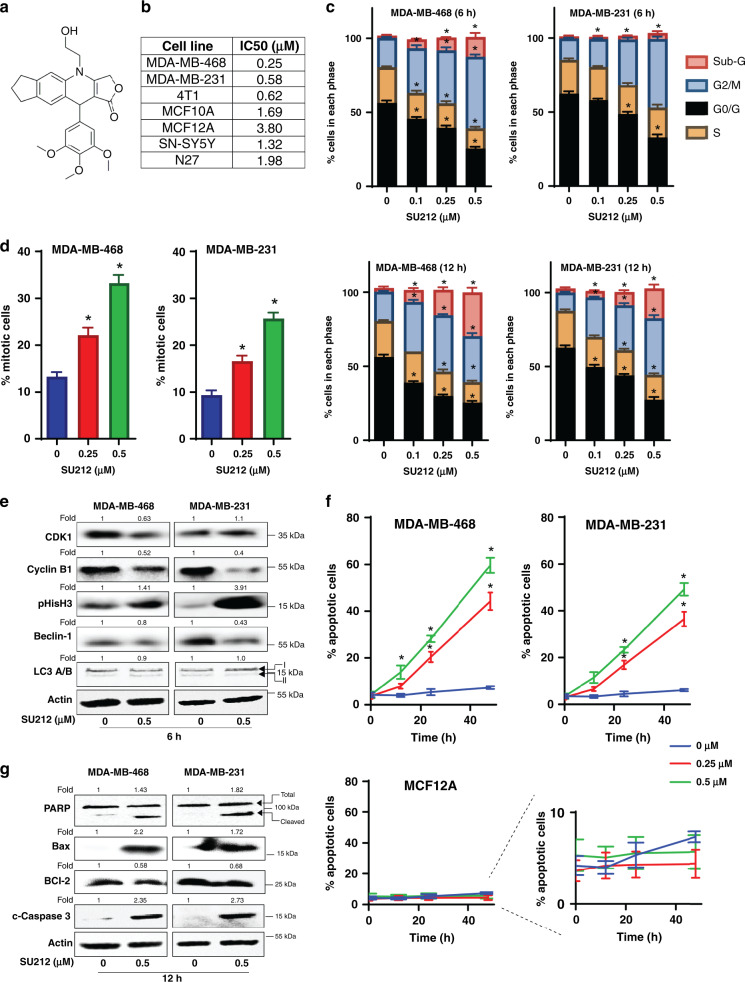
SU212 treatment induces mitotic phase arrest and apoptotic cell death in TNBC cells. **a** Chemical structure of SU212. **b** The growth-inhibitory effect SU212 was evaluated using MTT assay. In total, 5000 cells from TNBC cell lines (MDA-MB-231, MDA-MB-468 and 4T1), normal breast cell lines (MCF10A and MCF12A) and neural cell lines (SN-SY5Y and N27) cells were plated per well in a 96-well plate, and the next day, treated with vehicle (DMSO) alone or 0.01–10 µM of SU212 in fresh medium. After 48 h of these treatments, cell viability was recorded using MTT assay. **c**, **d** Effect of SU212 on cell- cycle distribution and cell-cycle regulators in human TNBC cells. **c** MDA-MB-468 and MDA-MB-231 cells were treated with vehicle or 0.1–0.5 µM concentrations of SU212 for 6 and 12 h. At the end of treatment, cells were collected and analysed for cell-cycle-phase distribution as detailed in the “Methods” section. **d** Percentage of mitotic cells in MDA-MB-468 and -231 cell cultures treated for 12 h with vehicle or 0.1–0.5 µM of SU212. At the end of treatment, cells were stained with Alexa Fluor 488 p-Histone H3 and PI and analysed using flow cytometry. **e** TNBC cells were treated with SU212 for 6 h, and total cell lysates were prepared as described in the “Methods” section. SDS-PAGE and western blot analysis were performed for cell-cycle and autophagic markers. Membranes were stripped and re-probed with anti-beta-actin antibody to ensure equal protein loading. **f** The apoptotic effect of SU212 on MDA-MB-468, MDA-MB-231 and MCF12A cells. Cells were treated with 0.25 or 0.5 µM SU212 for 12, 24 and 48 h. At the end of treatment, all cells were collected and stained with annexin V/PI and analysed for apoptotic cell population as mentioned in “Methods”. **g** TNBC cells were treated with SU212 for 12 h, and total cell lysates were prepared as described in “Methods”. SDS-PAGE and western blot analysis were performed for apoptotic cell death markers. Membranes were stripped and re-probed with anti-beta-actin antibody to ensure equal protein loading. Numbers on top of the bands represent changes in protein levels as determined by densitometric analysis of the immunoreactive bands, and corrected for beta-actin loading control. The cell number data are shown as mean ± SD of three independent plates; each sample was counted in duplicate. Data were analysed using a one-way ANOVA Dunnett’s test. * indicates *P* < 0.05.

An aza-podophyllotoxin derivative named SU212 (Fig. 1a) was synthesised and characterised as described previously.^8^ For detailed synthesis, see Supplementary Information.

### Cell culture

All cell lines used in the study were obtained from ATCC (Manassas, VA, USA). MDA-MB-231, MDA-MB-468 and 4T1 cell lines were grown in DMEM media (Corning, USA), and SN-SY5Y and N27 cell lines were grown in DMEM:F12 media (Corning, USA) supplemented with 10% FBS (Corning, USA) and 1% penicillin/streptomycin/Amphotericin B (Gibco, USA) in 95% air, 5% carbon dioxide at 37 °C. Normal fibrocystic breast epithelial cell lines MCF12A and MCF10A were grown in MEBM media (Lonza, Walkersville, MD) in 95% air, 5% carbon dioxide at 37 °C.

### Cell-viability assays

Cell viability was assessed using the MTT assay. In total, 5000 cells were plated in each well of 96- well plates and allowed to adhere for 24 h. Cells were treated with the respective concentration of SU212 (0.01, 0.1, 0.25, 0.5, 1 and 10 μM) and incubated for the 48-h time periods. Media was discarded, and 50 μL of MTT reagent (5 mg/ml in 1× PBS) (Millipore-Sigma, Burlington, MA) was added to each well followed by 1–2-h incubation. After incubation, MTT reagent was removed carefully without disturbing the MTT formazan crystals, which were then dissolved by adding 100 μL of DMSO. Absorbance was measured at 570 nm using a multimode plate reader. IC_50_ values were calculated using GraphPad Prism software.

### Cell-cycle phase-distribution analysis

The effect of SU212 treatment on cell-cycle distribution was evaluated by propidium iodide staining followed by flow cytometry analysis as previously described by Tailor et al.^9^ For details, see the Supplementary Information.

### Mitotic phase-arrest assay

Cells were plated at 30,000 cells/well of 12-well plates and treated with 0.25 or 0.5 μM of SU212 for 12 h. Following treatment, cells were trypsinised and pelleted. Cells were then incubated with Alexa Fluor 488 anti-p-Histone H3 antibody diluted in 1:500 ratio in FACS buffer (0.2% FBS in 1× PBS). Cells were incubated at 37 °C for 30 min. Cells were centrifuged and stained with a PI cocktail, and analysed using flow cytometry as described above in the cell-cycle-analysis assay.

### Apoptosis assay

Apoptotic cell death induction in MDA-MB-468, MDA-MB-231 and MCF12A cells in the presence of 0.25 or 0.5 μM SU212 at different time points was quantified using the Annexin V-FITC Apoptosis Assay kit from BioLegend, USA (Catalog number: 640914), using the manufacturer’s protocol.

### Western blot analysis

Total protein from vehicle and SU212-treated cells was analysed using immunoblotting as described by us previously.^10^ For detailed methods and antibody information, see the Supplementary Information.

### Lactate-production assay

Lactate production by MDA-MB-468, MDA-MB-231 and MCF12A cells in presence of 0.25 or 0.5 μM SU212 at different time points was quantified using the Lactate-Glo™ Assay kit from Promega, USA (Catalog number: J5021) according to the manufacturer’s protocol.

### ADP/ATP ratio assay

The ADP/ATP ratio in MDA-MB-468 and -231 cells in the presence and absence of SU212 (0.25 or 0.5 μM) after 1 and 6 h was assayed using ADP/ATP ratio assay kit from Sigma-Aldrich, USA (Catalog number: MAK135-1KT) by following the manufacturer’s protocol.

### Oil Red O staining

Oil Red O staining was performed as previously described by Nambiar et al.^5^ For details, see the Supplementary Information.

### Proteomic analysis

Tandem mass tag (TMT)-labelled total proteins from vehicle and SU212-treated cells were analysed using mass spectrometry, as previously described by us.^10^ For detailed information, see Supplementary Information. The mass spectrometry proteomics data have been deposited to the ProteomeXchange Consortium via the PRIDE^11^ partner repository with the dataset identifier PXD018929.

### In vivo tumour xenograft study

Animal studies were approved by Stanford University’s Institutional Animal Care and Use Committee (APLAC number: 32766).

#### MDA-MB-231 xenograft mouse model

In total, 7–8-week-old female NOD/SCID mice were injected in the right flank with 2 × 10^6^ MDA-MB-231 cells mixed with Matrigel (1:1). When tumour size reached approximately 200 mm^3^, the mice were randomly divided into three groups and the respective treatments were given. SU212 (15 and 30 mg/kg) was given in 20% PEG-300 (w/v) prepared in normal saline to animals as intraperitoneal injection continued for 7 days/week.

#### 4T1 xenograft mouse model

In total, 7–8-week-old, female Balb/c mice were injected in the right flank with 5 × 10^5^ 4T1 cells mixed with Matrigel (1:1). When tumour size reached ~50–100 mm^3^, the mice were randomly divided into two groups and the respective treatments were given. SU212 (30 mg/kg) was given in 20% PEG-300 (w/v) prepared in normal saline to animals as intraperitoneal injection continued for 7 days/week.

Tumour size was measured using a calliper, and tumour volume was calculated by the formula: 0.5236 × L1 × (L2)^2^, where L1 is the long diameter, and L2 is the short diameter. Mice were sacrificed on day 22.

Blood samples from anaesthetised (4% isoflurane in oxygen) animals were collected retro-orbitally. Blood plasma samples were analysed for different biochemical markers using Siemens Dimension Xpand analyser. At the end of each study, deeply anaesthetised mice were euthanised by cervical dislocation, and tumours and organs were collected.

### Lung-metastasis assay (tail vein)

In total, 1 × 10^6^ MDA-MB-231 cells were injected in 100 μL of 1× PBS into the lateral tail vein of 6–7-week-old female NOD/SCID mice. After 7 days of cell injection, each mouse was treated either with vehicle or 30 mg/kg SU212 every day for 4 weeks. At the end of the treatment, deeply anaesthetised mice were euthanised by cervical dislocation, and the lungs were dissected. Metastatic nodules were counted for each lung under a dissecting microscope. Lungs were fixed in neutral buffered formalin and further processed and embedded in paraffin. Paraffin-embedded tissue sections were stained with H&E.

### Immunohistochemical analysis of tumours

At the end of treatment, mice were euthanised, and tumours were fixed in neutral buffered formalin and embedded in paraffin. Paraffin-embedded tissue sections were deparaffinised and stained using a specific primary antibody followed by secondary antibody and Dab staining using ImmPACT^TM^ DAB kit (Vector Labs, CA). The biotinylated secondary antibodies used were horse anti-mouse IgG (Vector Labs, CA) and horse anti-rabbit IgG (Vector Labs, CA). The percentage of Ki-67-positive cells, Bax, p-AMPKα, c-Caspase 3 and lactate dehydrogenase A (LDHA) expression was quantified by counting brown-stained cells within the total number of cells at five arbitrarily selected fields from each tumour.

### Statistical analysis

Statistical analyses for each experiment were performed using GraphPad Prism 6.0 software. Quantitative data in each figure are presented as mean ± SD, where * indicates *P* < 0.05, ** indicates *P* < 0.01 and *** indicates *P* < 0.001. Statistical significance of the difference between control and the treated group was determined by the Student’s *t* test and one-way ANOVA followed using Dunnet’s adjustment.

## Results

### SU212 induces mitotic phase arrest and apoptotic cell death in TNBC cells

The in vitro effect of SU212 treatment on TNBC cells (MDA-MB-468, MDA-MB-231 and 4T1) and normal breast cell lines (MCF10A and MCF12A) was assessed by MTT assay (Fig. 1b). IC_50_ values for MDA-MB-468 and MDA-MB-231 cells were 0.25 and 0.58 μM after 48 h of treatment, respectively. Whereas IC_50_ values for treatment of normal breast cell lines for 48 h were 1.69 and 3.8 μM for MCF10A and MCF12A cells, respectively (Fig. 1b). Treatment with 0.1–0.5 μM of SU212 appears to have minimal effects on normal breast cell lines even after 72 h of treatment. Based on these data, we selected 0.1–0.5 μM for further studies. As a side effect, etoposide induces neurotoxicity and peripheral neuropathy. To test whether SU212 induces any neurotoxicity, we have tested its effect on cell viability of neuronal cell Lines (SH-SY5Y and N27). IC_50_ values for treatment of neuronal cell lines for 48 h were 1.32 and 1.98 μM for SH-SY5Y and N27 cells, respectively (Fig. 1b).

Propidium iodide (PI) staining for cell-cycle analysis showed that 6 and 12 h of treatment with SU212 induces G2-/M-phase arrest in both TNBC cell lines (Fig. 1c). This arrest was followed by an increase in the sub-G1 phase (20–35%, *P* < 0.05) with 6-h treatment. Cells were also stained with phospho-Histone H3 and PI to differentiate between G2- and M-phase arrest. These experiments showed that SU212 treatment at 6-h time point induces mitotic phase arrest (20–31%, *P* < 0.05) in both TNBC cell lines (Fig. 1d). This result was further confirmed by western blot analysis of G2-/M- phase regulators (Fig. 1e). Proteomic analysis and GSEA revealed that SU212 treatment decreases the abundances of different forms of tubulin, which may also lead to the observed mitotic phase arrest (Supplementary Fig. S1B). Cyclins and cyclin-dependent protein kinases (CDKs) are the key drivers of the eukaryotic cell cycle and associated with the regulation of cell division. Treatment with SU212 (0.5 μM) for 6 h resulted in the downregulation of cyclin B1 and CDK1 expression, and an increase in the expression of phospho-histone H3, which explains the G2/M-transition cell-cycle arrest induced by SU212 (Supplementary Fig. S1A). Prolonged arrest in the mitotic phase resulting from SU212 treatment eventually leads to increased apoptosis. Annexin-V staining revealed that treatment with SU212 for 12–48 h induces 12–60% (*P* < 0.05) apoptotic cell death in TNBC cell lines, in contrast to negligible change in apoptosis in the normal cell line MCF12A (Fig. 1f). This was further confirmed by western blot analysis of apoptosis regulators, including cleaved PARP, Bax, Bcl-2 and cleaved caspase 3. SU212 treatment for 12 h induced pro-apoptotic Bax expression and inhibited Bcl-2 expression, leading to a significant increase in Bax/Bcl-2 ratio that determines cell susceptibility to undergo apoptosis (Fig. 1g and Supplementary Fig. S1C, D). SU212 treatment also induces the cleavage of PARP and caspase 3 (Fig. 1g and Supplementary Fig. S1C). To differentiate between apoptotic and autophagic cell death, we blotted Beclin-1 and LC3 A/B and found that SU212 treatment inhibits Beclin-1 but did not affect LC3 A/B (Fig. 1e and Supplementary Fig. S1A). This result confirms that SU212 treatment does not induce autophagic cell death (Fig. 1e).

### Treatment with SU212 causes energy stress-independent AMPK activation and blocks lipogenesis in TNBC cells

**Fig. 2:**
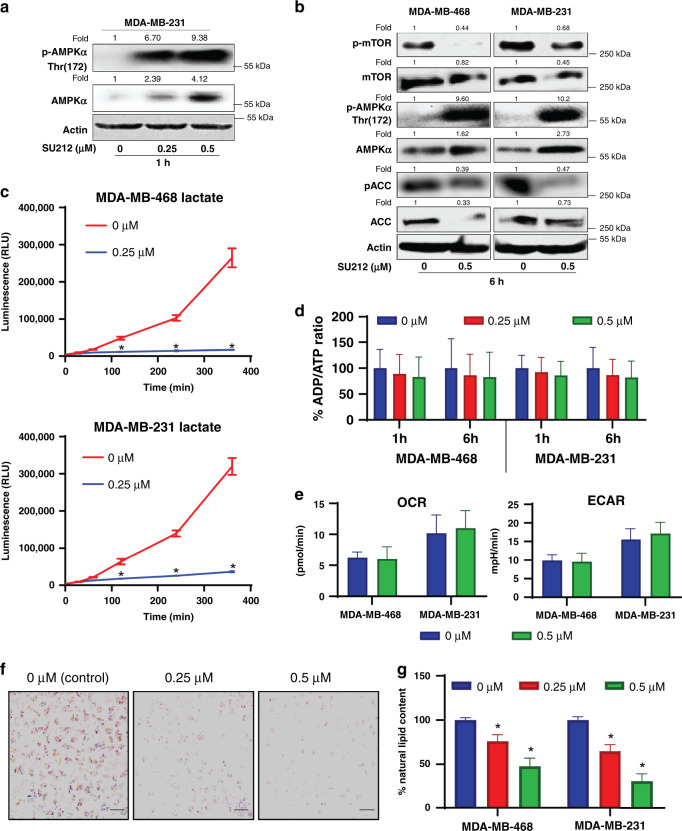
SU212 treatment leads to AMPK activation and inhibits lipogenesis. **a**, **b** Total cell lysates were prepared as described in “Methods”. SDS-PAGE and western blot analysis were performed for AMPK signalling proteins. Membranes were stripped and re-probed with anti-beta-actin antibody to ensure equal protein loading. **a** Immunoblotting of total and phospho-AMPKα for a total cell lysate of MDA-MB-231 prepared after 1-h treatment of SU212. **b** Immunoblotting of proteins associated with the AMPK pathway from a total cell lysate of MDA-MB-468 and MDA-MB-231 prepared after 6-h treatment of SU212. **c** Lactate-production assay. Both TNBC cell lines were treated with SU212, and lactate production was measured at different time points starting from 30 to 360 min. **d** Quantification of the ADP/ATP ratio. MDA-MB-468 and MDA-MB-231 cells were treated with 0.5 µM SU212, and total cellular ATP was quantified as described in “Methods”. **e** Oxygen-consumption rate (OCR) and extracellular acidification rate (ECAR) measurement in vehicle and SU212 (0.5 µM)-treated TNBC cells. Cells were treated with SU212, and after 1 and 6 h, OCR and ECAR were measured using Seahorse XF96 Extracellular Flux Analyser. **f**, **g** Equal number of both TNBC cells were plated and analysed for neutral lipids by Oil red O (ORO) staining as detailed in “Methods”. **f** Representative images of cells stained with ORO (at ×20), scale bar, 125 μm, and **h** quantification of ORO staining in human TNBC cells following SU212 (0.25 and 0.5 μM) treatment for 6 h. Quantification data for ORO staining were normalised with respect to the cell number for each group. Data shown were mean ± SD of three independent plates; each sample was counted in duplicate. Data were analysed using one-way ANOVA Dunnett’s test. * indicates *P* < 0.05, significantly different compared with the corresponding control.

Our experiments showed that SU212 activated AMPK via phosphorylation of AMPKα at Thr172 within 1 h of treatment (Fig. 2a) in MDA-MB-231 cell lines. SU212 (0.5 μM) treatment for 6 h induced robust activation of AMPKα (Fig. 2b and Supplementary Fig. S2A, B). This was followed by downregulation of mTOR and acetyl-CoA carboxylase (ACC) inhibition (Fig. 2B and Supplementary Fig. S2A). Since AMPK activation negatively regulates the Warburg effect, we measured the levels of lactate produced by the cells post SU212 treatment. As anticipated, treatment with SU212 (0.25 μM) for 30 min–6 h inhibited lactate production in both TNBC cells (Fig. 2c).

AMPK activation can occur in the presence of energy stress. To verify the activation of AMPK and its energy stress dependence, cellular ATP levels were measured in the absence and presence of SU212 at different time points. SU212 treatment (0.5 μM) at 1–6 h did not affect total cellular ATP levels in TNBC cell lines (Fig. 2d). The result was further confirmed via C13-glucose-tracking assay. MDA-MB-231 cells were fed C13-labelled glucose and lysed at different time points. Samples were analysed by LC–MS/MS for C13 tracing. SU212 treatment for 1–6 h did not affect the cellular level of d-glucose, glucose-6-phosphate/fructose-6-phosphate, ATP, citrate and α-ketoglutarate (Supplementary Fig. S3). To determine if AMPK was activated under hypoxic stress, the oxygen- consumption rate (OCR) and extracellular acidification rate (ECAR) were measured in the presence of SU212 using a Seahorse XF96 Extracellular Flux Analyzer. Data suggest that SU212 treatment for 1 and 6 h did not affect the OCR and ECAR in both TNBC cell lines (Fig. 2e). Also, SU212 treatment did not cause energy stress at an early time point. Taken together, these results suggest that AMPK activation by SU212 is not dependent on either energy or hypoxic stress.

Lipogenesis is the key event by which acetyl-CoA is converted into triglycerides for the storage of fat. AMPK activation not only affects protein synthesis via mTORC1 inhibition, but also inhibits lipogenesis. As noted above, SU212 treatment inhibits ACC. Therefore, we further evaluated the effect of SU212 treatment on the total cellular lipid accumulation. Both TNBC cell lines were stained with oil red O (ORO) to measure for triglyceride content. We found that treatment with SU212 (0.25 and 0.5 μM) for 6 h decreased the cellular lipid content by 24–70% (*P* < 0.05) in both TNBC cell lines (Fig. 2f, g), further indicating the AMPK activation by SU212.

### SU212 induces oxidative phosphorylation and decreases glycolysis in TNBC cells

**Fig. 3:**
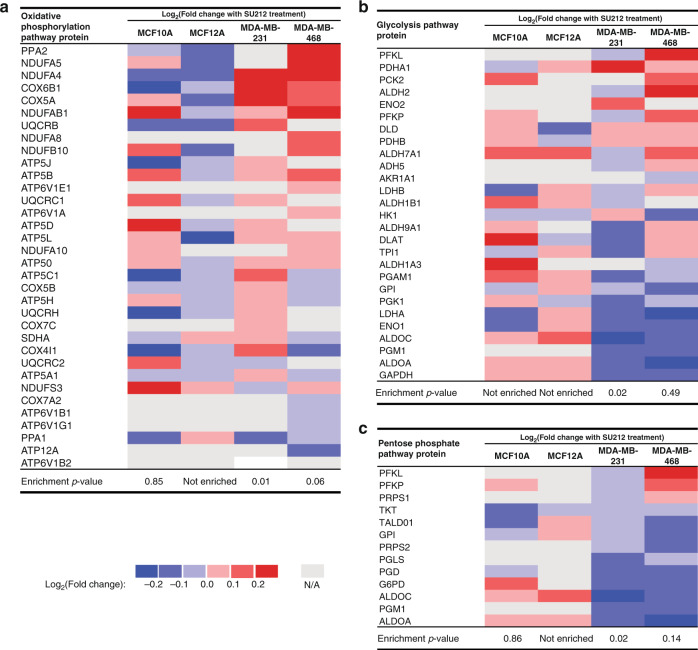
GSEA was performed on the proteomics results to determine the enrichment of KEGG pathways upon treatment of MCF10A and MCF12A or TNBC MDA-MB-231 and MDA-MB-468 cell lines with SU212 (12 h, 0.5 μM). **a** Enrichment in the oxidative phosphorylation pathway was observed in proteins that increase in abundance upon treatment with SU212 for TNBC cell lines. **b** Enrichment in the glycolysis and **c** pentose phosphate pathways was observed in proteins that decrease in abundance upon treatment with SU212 for TNBC cell lines.

To better understand the broader effect of SU212 (0.5 μM, 12 h) on TNBC and normal breast cell lines, quantitative proteomic analysis was performed on proteins from lysates of treated breast cancer and normal cell lines. KEGG pathway analysis revealed a significant increase in the levels of proteins associated with oxidative phosphorylation upon TNBC treatment with SU212 (enrichment *P* values of 0.01 and 0.06 for MDA-MB-231 and MDA-MB-468 cell lines, respectively, Fig. 3a). Conversely, a decrease in the levels of proteins associated with glycolysis (Fig. 3b) and the pentose phosphate pathway (Fig. 3c) was observed upon treatment of TNBC cell lines with SU212 (enrichment *P* values of 0.02 and 0.49 for glycolysis in MDA-MB-231 and MDA-MB-468 cell lines, respectively, and *P* values of 0.02 and 0.14 for the pentose phosphate pathway in MDA-MB-231 and MDA-MB-468 cell lines, respectively). In normal breast cell lines, alterations in these pathways were not significant (Fig. 3a–c). These results suggest that SU212 specifically activates AMPK signalling in TNBC cell lines but not in normal breast cell lines.

### Cytotoxic effect of SU212 is dependent on AMPK activation

**Fig. 4:**
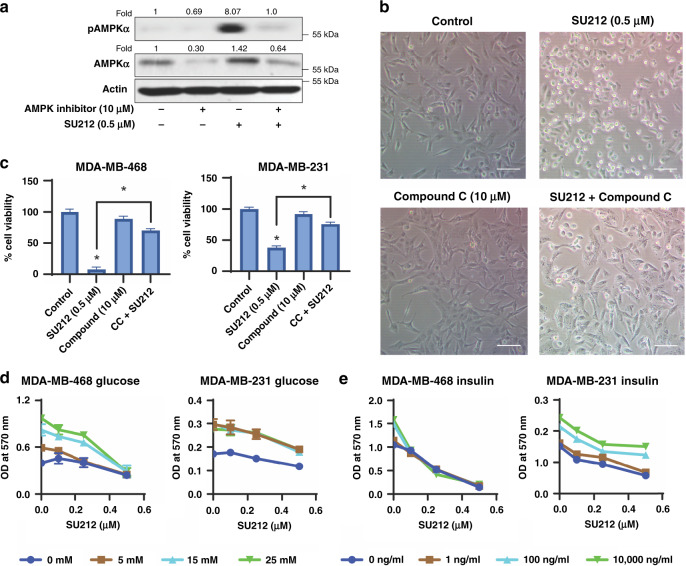
SU212 induces cytotoxicity in TNBC cells via AMPK activation. TNBC cells were pre-treated with a specific AMPK inhibitor Compound C (CC) followed by vehicle or SU212 treatment in the same media. **a** Immunoblotting of total and phospho-AMPKα for a total cell lysate of MDA-MB-231 prepared after 6-h treatment with CC, SU212 or their combination. **b** Microscopic observation of MDA-MB-231 cell morphology at ×10 magnification. Scale bar, 250 μm. **c** In total, 5000 TNBC cells were plated in 96-well plates and treated with vehicle, CC (10 µM), SU212 (0.1, 0.25 and 0.5 µM), or their combination for 72 h. Cell viability was assayed using the MTT assay as described in the Methods section. **d** Effect of SU212 treatment on TNBC cell viability under different glycaemic conditions. **e** Effect of SU212 treatment on TNBC cell viability under different insulin conditions. Data shown were the mean ± SD of three independent plates; each sample was counted in duplicate. Data were analysed using one-way ANOVA Dunnett’s test. * indicates *P* < 0.05.

Compound C (CC) is a cell-permeable selective and reversible inhibitor of AMPK. To determine if SU212 depends on AMPK for its cytotoxic effect, CC was used to treat TNBC cell lines and test if treatment inhibits expression of AMPK and reverses the subsequent effects of SU212 observed in these cells. Cells were pre-treated (2 h) with CC prior to SU212 treatment in the same media. Cell lysates were prepared 6 h post SU212 treatment and analysed for phospho-AMPKα (Thr172) and total AMPKα levels. The results suggest that CC treatment at 10 μM concentration completely inhibits phospho-AMPKα (Thr172) as well as expression of total AMPKα (Fig. 4a–c). However, SU212 treatment in the presence of CC did not induce AMPK (Fig. 4a). Microscopic observation at ×10 magnification suggests that co-treated cells were healthier and had a morphology similar to vehicle-treated cells (Fig. 4b). After 72 h of co-treatment, the cell viability of both TNBC cell lines was assessed. Data show that pre-treatment of CC reverts the (*P* < 0.001) cytotoxic effect of SU212 by 73–86% (Fig. 4c). These results strongly suggest that AMPK activation is the major target of SU212 cytotoxicity in TNBC cell lines.

### SU212 activates AMPK independent of energy stress in TNBC cell lines

The availability of glucose or differential glycaemic state can modulate the expression of AMPK inside cells. To evaluate the effect of different glycaemic states on SU212’s inhibitory effect, TNBC cell lines were treated with media containing 0, 5, 15 or 25 mM glucose. Treatment with SU212 inhibited the cell viability in MDA-MB-468, which was unaltered by glycaemic state (Fig. 4d). However, in MDA-MB-231 cells, viability assays suggest that hypoglycaemic conditions provide an additive effect with SU212 (0.1–0.5 μM) treatment and accounted for around 20–30% (*P* < 0.05) improvement in inhibition compared to hyperglycaemic conditions (Fig. 4d).

Insulin signalling activates the phosphoinositide 3-kinase (PI3K)–AKT, mitogen-activated protein kinases (MAPK) and mTOR-signalling pathways in cells. This leads to lipogenesis, protein synthesis, cell proliferation and therapy resistance in cancer cells. The insulin-induced PI3K pathway is also associated with AMPK inhibition. To show that AMPK activation is independent of insulin levels, we tested the effect of SU212 on cells cultured under hyper-insulin conditions. Physiologically, 100 ng/ml insulin is considered to be a hyper-insulin condition.^7^ During these experiments, 1 ng/ml (normal physiological concentration), 100 ng/ml (hyperinsulinaemia) and 10,000 ng/ml (to ensure the activation of insulin signalling at the highest threshold) were used.^7^ These experiments show that insulin did not affect the cytotoxicity of MDA-MB-468 cells when treated with SU212 (Fig. 4e). However, 10,000 ng/ml insulin did induce resistance to SU212 treatment in MDA-MB-231 cells as evidenced by 17% reduction in cytotoxicity (Fig. 4e). Cells with 1 and 100 ng/ml insulin did not show any significant difference in cytotoxicity of SU212 (Fig. 4e).

### SU212 treatment inhibits tumour progression and metastasis in a xenograft mouse model

**Fig. 5:**
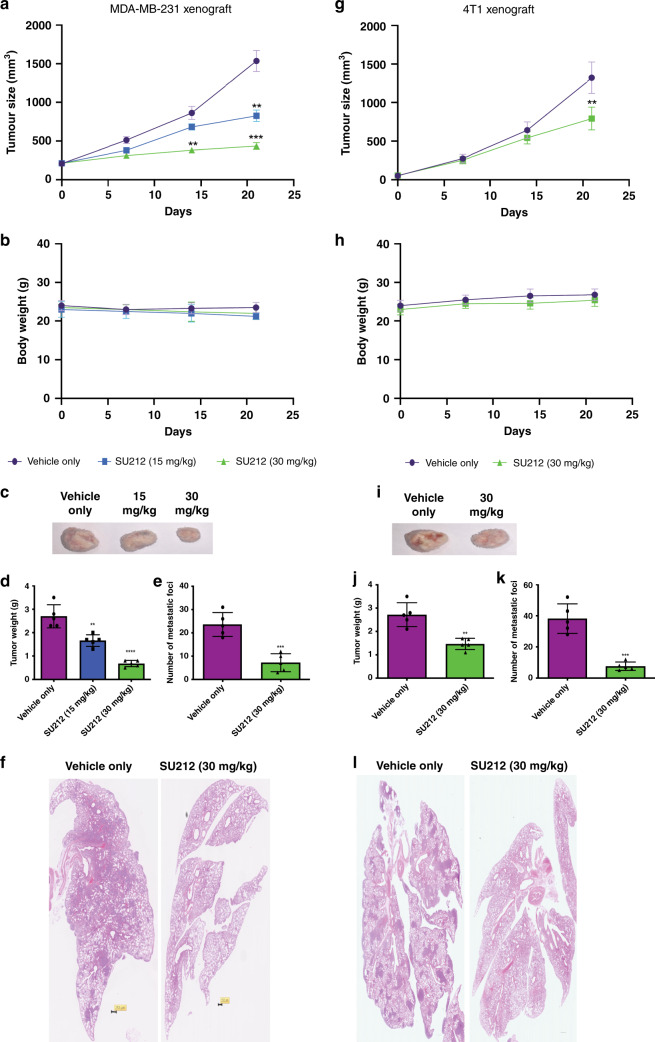
SU212 treatment inhibits the tumour progression and metastasis. **a**–**f** In all, 2 × 10^6^ MDA-MB-231 cells were implanted subcutaneously in the right flank of female NOD-SCID mice. Mice were randomised and divided into three groups (*n* = 5). Each group was treated with vehicle or 10 mg/kg or 30 mg/kg SU212 for 21 days. **a** Tumour volumes were measured using callipers for each group’s tumours (*n* = 5). **b** Mouse body weight was recorded at regular intervals during treatment. **c** Representative image of excised tumours at the end of treatment. **d** Excised tumour weights. **e**, **f** SU212 treatment inhibited lung metastasis in MDA-MB-231 tail-vein lung-metastasis model. In all, 1 × 10^6^ cells were injected via the tail vein in female NOD-SCID mice (*n* = 5). Each group of mice was treated with either vehicle or 30 mg/kg SU212 for 30 days. **e** Each lung was scored for metastatic foci under a dissecting microscope. **f** Representative images of H&E-stained lung section. Scale bar, ~250 μm. **g**–**k** In total, 5 × 10^5^ 4T1 cells were implanted subcutaneously in the right flank of female Balb/c mice. Mice were randomised and divided into two groups (*n* = 5). Each group was treated with vehicle or 30 mg/kg SU212 for 21 days. **g** Tumour volumes for each group’s tumours (*n* = 5). **h** Mouse body weight. **i** Representative images of excised tumours at the end of treatment. **j** Excised tumour weights. **k**, **l** At the end of treatment, the lung from each mouse was analysed for spontaneous lung metastasis from subcutaneous 4T1 tumour. **k** Each lung was scored for metastatic foci under a dissecting microscope. **l** Representative images of H&E-stained lung cessation. Scale bar, ~200 μm. Data were analysed using Student’s *t* test. Data are shown as the mean ± SD of five mice; *P < 0.05, **P < 0.01 and ***P < 0.001 significantly different compared with the corresponding control.

To determine the in vivo effects of SU212 treatment on tumour progression, a study was performed using luciferase-labelled MDA-MB-231 xenograft mouse model. Mice bearing tumours of 150–200 mm^3^ were randomised and treated with either vehicle or SU212 (15 or 30 mg/kg) (*n* = 5) intraperitoneally (i.p.) for 21 days. Tumour sizes were measured using vernier calliper (Fig. 5a). Consistent with the in vitro data, treatment with SU212 in 15 mg/kg and 30 mg/kg doses inhibited TNBC tumour growth by 46 and 71%, respectively (Fig. 5a). During 21 days of treatment, mice did not show significant body-weight changes (Fig. 5b), and no stress or pain behaviour was observed. Following treatment, mice were euthanised, tumours were collected (Fig. 5c) and tumour weight was recorded (Fig. 5d). Tumours from mice that received 15 mg/kg and 30 mg/kg doses of SU212 had 42 and 81% less tumour weight, respectively, compared to tumours from untreated mice (Fig. 5d).

To evaluate the effect of SU212 on lung metastasis, a tail-vein lung-metastasis mouse model was used. MDA-MB-231 cells were injected into the tail vein, and mice were treated with either vehicle or SU212 (30 mg/kg) for 30 days. At the end of treatment, mice were euthanised, and lungs were collected for counting metastatic foci. Fixed lungs were stained, and H&E staining showed that treatment with SU212 reduced the number of metastatic foci in the lung by 69% (Fig. 5e) compared to control. This result suggests that SU212 treatment possibly inhibits lung metastasis via inhibiting cell proliferation and inducing cell death in tumour cells.

To confirm this further, we also used the 4T1 syngeneic mouse xenograft that spontaneously metastasises to the lung and serves as a model to study the effect of treatment on tumour progression and metastasis, simultaneously. Mice bearing 50–100-mm^3^ tumour were randomised and treated with either vehicle or SU212 (30 mg/kg) (*n* = 5) intraperitoneally (i.p.) for 21 days. Consistent with the MDA-MB-231 xenograft and tail-vein lung-metastasis mouse model, treatment with SU212 inhibited tumour growth by 40% (Fig. 5g) without significant body-weight loss (Fig. 5h). Tumour weight of SU212-treated group was 46% less compared to control (Fig. 5I, j). Lung analysis at the end of treatment followed by H&E staining also revealed that SU212 treatment reduces the number of metastatic foci in the lung by 80% compared to control (Fig. 5k, l).

**Fig. 6:**
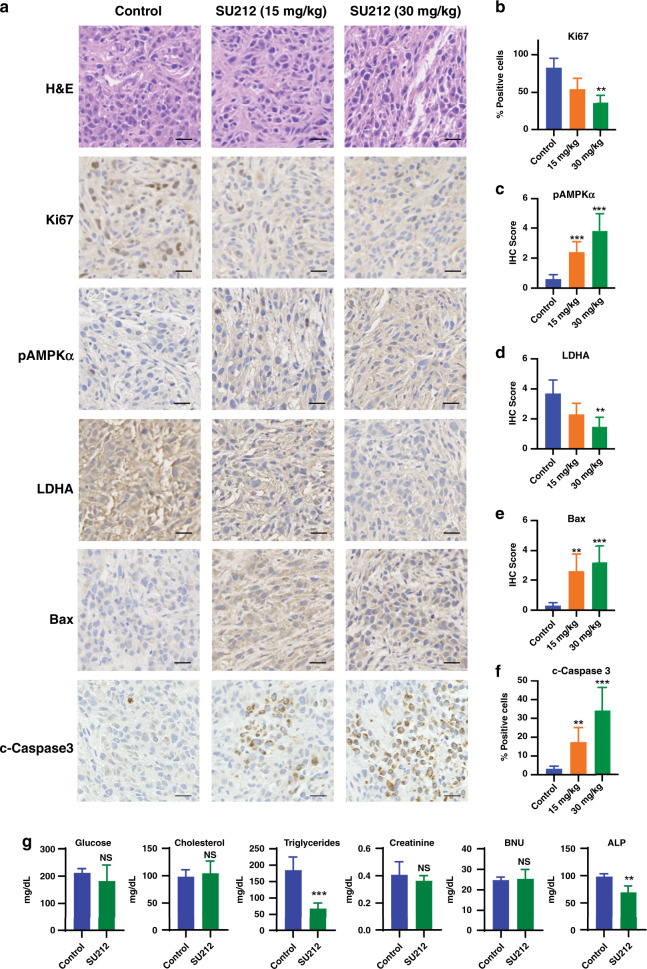
SU212 treatment reduced expression of cell proliferation markers and enhanced apoptosis in MDA-MB-231 xenograft. Tumour xenograft tissue samples were fixed and immunohistochemically analysed for Ki-67, p-AMPKα, LDHA, Bax and cleaved Caspase 3-positive cells as detailed in the “Methods” section. **a** The representative pictographs (×40 magnification, scale bar, 50 μm) for positive brown-stained cells for each of the markers are shown from control (vehicle only) and treatment groups. Quantitative data for **b**, Ki-67, **c**, p-AMPKα, **d**, LDHA, **e**, Bax and **f**, c-Caspase 3 from each mouse in each group. Percentage of positive cells and IHC score is relative to the control. **g** Blood samples were collected from each mouse (control (vehicle only) and SU212 treated (30 mg/kg)), and serum was analysed for the levels of glucose, cholesterol, triglycerides, creatinine, BNU and alkaline phosphatase (ALP) on a Siemens Dimension Xpand Plus HM analyser. Data were analysed using Student’s *t* test. Data are shown as the mean ± SD of five mice. *P < 0.05, **P < 0.01, ***P < 0.001, NS not significant when compared with the corresponding control.

Tumours were fixed and sectioned for immunohistochemical (IHC) analyses. Tumour sections were stained for Ki-67, Bax, phospho-AMPKα (Thr172), LDHA, cleaved caspase 3 and H&E staining (Fig. 6a). We found that SU212 treatment inhibited the expression of Ki-67 and LDHA, and induced the expression of p-AMPKα, Bax and c-Caspase 3 (Fig. 6b–f). These results are consistent with the in vitro data (Fig. 6b–f). Moreover, in vivo treatment induced the expression of p-AMPKα, which further confirmed that SU212 treatment causes an inhibition of tumour progression via the AMPK pathway. Furthermore, Ki-67 is a cell proliferation marker, and SU212-treated tumours had less Ki-67 expression compared to vehicle-treated tumours, indicating that treatment with SU212 inhibits tumour cell proliferation. Treatment with SU212 inhibited the expression of LDHA, which is an indicator of the Warburg effect due to its role in aerobic glycolysis and lactate production by cancer cells. Also, SU212 induced the expression of Bax and cleaved caspase 3, consistent with western blot and apoptosis assays (Fig. 6e, f).

Blood serum was collected at the end of treatment from each mouse, and levels of blood glucose, cholesterol, triglycerides, alkaline phosphatase (ALP), creatinine and blood urea nitrogen (BUN) were measured. Treatment with SU212 (30 mg/kg) for 21 days did not affect blood glucose, cholesterol, creatinine and BUN, whereas levels of triglycerides and ALP decreased significantly (Fig. 6g). Constant blood glucose levels further suggest that the effect of SU212 is independent of energy stress. Reduced levels of triglycerides also support in vitro data, indicating that treatment with SU212 inhibits lipogenesis. The level of ALP is an indicator of liver toxicity, and creatinine and BUN are indicators of nephrotoxicity. Thus, the results from these studies confirm that SU212 treatment did not cause liver- or nephrotoxicity.

## Discussion

Studies of podophyllotoxins, the natural compounds from *Podophyllum peltatum*, have led to numerous pioneering advances, including the development of well-known anticancer drugs,^12^ including etoposide,^13^ which also causes side effects. However, in combination with other small-molecule anticancer drugs, high doses of etoposide have remained an effective treatment strategy for refractory Hodgkin’s lymphoma, non-Hodgkin’s lymphoma, acute leukaemia and other refractory haematological cancers. Success of podophyllotoxin derivatives has motivated researchers to develop similar anticancer drugs with higher potency and less toxicity.^14^ Several modifications in the core chemical structure of podophyllotoxin have been attempted to find a better drug. For example, a nitrogen heteroatom at the C4 position was incorporated into the etoposide structure,^15,16^ and C4-nonsugar derivatives of etoposide (e.g., aza-podophyllotoxin) have been investigated for their anticancer properties.^17–22^

Earlier work from our group leads to the discovery of novel derivatives of etoposide that showed significant anticancer activity.^23^ Herein, we are reporting the results of our studies with an aza-podophyllotoxin compound SU212, which is found to be highly active against the triple-negative breast cancer cells.

Nearly a century ago, Otto Warburg suggested the role of metabolic reprogramming in favouring aerobic glycolysis in cancer cell proliferation, required for maintaining rapid cell growth and cell division.^24^ The importance of metabolic reprogramming in cancer cells is now well accepted as a hallmark of cancer.^25^ Moreover, metabolic syndromes, including type 2 diabetes, increase the risk of breast cancer by 27% due to hyperglycaemia and insulin resistance.^26^ AMP kinase (AMPK) is a master regulator and sensor of energy balance in the cell and, mostly found to be conserved during evolution.^27^ AMPK is activated under stress conditions, predominantly in response to energy stress due to an imbalance between ATP and AMP. AMPK suppresses high-energy-consuming cellular processes such as macromolecule synthesis by inhibiting mTORC1 and halting cell division. AMPK activation also shifts cellular metabolism from aerobic glycolysis to more energy-efficient oxidative phosphorylation.^28^ Therefore, AMPK is an attractive therapeutic target to inhibit cancer proliferation. Calorie restriction (CR) in cancer cells and to induce AMPK activity, several preclinical and clinical studies with various chemotherapeutic drugs have been performed. The approaches used to induce CR include dietary restriction (fasting) and using CR mimetic agents such as metformin.^29^ CR mimetic compounds are dependent on the cell’s glycaemic and insulin states, and have been shown to restrict the proliferation of TNBC cell lines in normoglycaemic conditions only, whereas TNBC cells cultured in hyperglycaemic conditions are resistant to metformin treatment.^7^ By contrast, AMPK activation, regardless of a cell’s glycaemic or insulin state, can inhibit cancer proliferation.^30^ In search of energy stress-independent (direct) activators of AMPK, we have developed this novel derivative of podophyllotoxin, SU212.

Unlike metformin and 2-deoxy-d-glucose (2-DG), SU212 treatment does not affect cellular ATP levels, glucose uptake or oxygen-consumption rate (OCR) during the early hours of treatment, whereas AMPK is activated within the first hour of treatment in TNBC cell lines, as confirmed by downstream inactivation of mTORC1. These data demonstrated that direct activation of AMPK occurs with SU212, irrespective of the energy state of TNBC cells. In addition, inhibition of mTORC1 by different chemotherapeutic agents such as rapamycin has been shown to enhance the therapeutic effects of other anticancer agents. Thus, it is possible that SU212 could also be used in synergy with other chemotherapeutics. The Compound C combination study confirmed the specificity of SU212 for AMPK and demonstrated that SU212 can decrease TNBC cell viability under both hyperglycaemic and hyper-insulin conditions. A hyperglycaemic state negatively regulates AMPK activity via influencing AMP:ATP ratio.^31^ A hyper-insulin state also inhibits AMPK activation via PI3K/Akt signalling that is known to inhibit AMPK.^32^ Our studies have shown that under both hyperglycaemic and insulin conditions, SU212 activates AMPK, which also supports its role as a direct activator of AMPK. SU212 is an analogue of etoposide that is known to cause DNA breaks.^33^ DNA damage/breaks also lead to energy stress-independent AMPK activation,^34^ and this could be a possible mechanism of AMPK activation by SU212.

Treatment of TNBC cell lines with SU212 inhibits cell proliferation by causing mitotic phase arrest and apoptotic cell death. This effect is mainly driven by AMPK activation. SU212 treatment induces AMPKα activation with mitotic phase arrest at 6 h and apoptotic cell death at 12–72 h. Although AMPK is associated with energy metabolism, it also plays a role during the G2/M phase via CDC25C and mitotic spindle assembly inhibition.^30,35^ Although AMPK activation and inhibition of mTORC1 leads to autophagy, SU212 treatment induces apoptotic cell death and inhibits the expression of Beclin-1. Therefore, a persistent arrest of cells in a mitotic phase may induce apoptotic cell death rather than autophagy. Similarly, mTORC1 plays a role in lipogenesis and induces lipid accumulation in cancer cells, which is also important in the development of treatment resistance.^5^ AMPK activation inhibits ACC, a precursor molecule for lipogenesis and SREBP1 that can inhibit lipid metabolism.^5,31^ Our results also suggest that SU212 treatment inhibits the accumulation of lipids in breast cancer cells via AMPK activation and ACC inhibition.

SU212 treatment was shown to inhibit tumour progression and metastasis in MDA-MB-231 and 4T1 xenograft and MDA-MB-231 tail-vein metastasis models. Furthermore, SU212 treatment increased the expression of p-AMPKα, Bax and c-Caspase 3, and inhibited the expression of Ki-67 and LDHA. While SU212 treatment did not affect blood glucose levels, which indicates that the effect of SU212 is independent of energy stress. SU212 treatment also reduced cellular triglycerides confirming lipogenesis inhibition. According to Genomics of Drug Sensitivity in Cancer database (https://www.cancerrxgene.org/compound/Etoposide/134/overview/ic50), the IC_50_ value of etoposide for MDA-MB-231 cells is 6.02 μM. However, through modification in the core chemical structure, we have been able to improve its activity by nearly tenfold. Etoposide induces energy stress-dependent AMPK activation and autophagy,^36^ whereas SU212 directly activates AMPK followed by apoptotic cell death. Importantly, SU212 treatment also does not show any liver- or nephron toxicity or weight loss, which is one of the detrimental effects observed with etoposide.

In summary, we have identified a potential new drug, i.e., SU212 that is highly potent and relatively safe. SU212 activates AMPK signalling independent of intracellular energy levels, followed by inhibition of lipogenesis and induction of apoptotic cell death. This study has provided a foundation for further development of SU212 as a clinical candidate for treatment of triple-negative breast cancer.

## Supplementary information

Supporting material

## Data Availability

The mass spectrometry proteomics data have been deposited to the PRIDE Archive (http://www.ebi.ac.uk/pride/archive/) via the PRIDE partner repository with the dataset identifier PXD018929.

## References

[1] Siegel RL, Miller KD, Jemal A (2018). Cancer statistics, 2018. CA Cancer J. Clin..

[2] Liberti MV, Locasale JW (2016). The Warburg effect: how does it benefit cancer cells?. Trends Biochem. Sci..

[3] Faubert B, Boily G, Izreig S, Griss T, Samborska B, Dong Z (2013). AMPK is a negative regulator of the Warburg effect and suppresses tumor growth in vivo. Cell Metab..

[4] Motoshima H, Goldstein BJ, Igata M, Araki E (2006). AMPK and cell proliferation-AMPK as a therapeutic target for atherosclerosis and cancer. J. Physiol..

[5] Nambiar DK, Deep G, Singh RP, Agarwal C, Agarwal R (2014). Silibinin inhibits aberrant lipid metabolism, proliferation and emergence of androgen-independence in prostate cancer cells via primarily targeting the sterol response element binding protein 1. Oncotarget.

[6] Howell JJ, Hellberg K, Turner M, Talbott G, Kolar MJ, Ross DS (2017). Metformin inhibits hepatic mTORC1 signaling via dose-dependent mechanisms involving AMPK and the TSC complex. Cell Metab..

[7] Zordoky BN, Bark D, Soltys CL, Sung MM, Dyck JR (2014). The anti-proliferative effect of metformin in triple-negative MDA-MB-231 breast cancer cells is highly dependent on glucose concentration: implications for cancer therapy and prevention. Biochim Biophys. Acta.

[8] Kim YS, Kumar V, Lee S, Iwai A, Neckers L, Malhotra SV (2012). Methoxychalcone inhibitors of androgen receptor translocation and function. Bioorg. Med. Chem. Lett..

[9] Tailor D, Hahm ER, Kale RK, Singh SV, Singh RP (2014). Sodium butyrate induces DRP1-mediated mitochondrial fusion and apoptosis in human colorectal cancer cells. Mitochondrion.

[10] Going CC, Tailor D, Kumar V, Birk AM, Pandrala M, Rice MA (2018). Quantitative proteomic profiling reveals key pathways in the anticancer action of methoxychalcone derivatives in triple negative breast cancer. J. Proteome Res..

[11] Perez-Riverol Y, Csordas A, Bai J, Bernal-Llinares M, Hewapathirana S, Kundu DJ (2019). The PRIDE database and related tools and resources in 2019: improving support for quantification data. Nucleic Acids Res..

[12] Eyberger AL, Dondapati R, Porter JR (2006). Endophyte fungal isolates from *Podophyllum peltatum* produce podophyllotoxin. J. Nat. Prod..

[13] Bohlin L, Rosen B (1996). Podophyllotoxin derivatives: drug discovery and development. Drug Discov. Today.

[14] Kobayashi K, Ratain MJ (1994). Pharmacodynamics and long-term toxicity of etoposide. Cancer Chemother. Pharm..

[15] Ren J, Wu L, Xin WQ, Chen X, Hu K (2012). Synthesis and biological evaluation of novel 4beta-(1,3,4-oxadiazole-2-amino)-podophyllotoxin derivatives. Bioorg. Med. Chem. Lett..

[16] Zhao Y, Ge CW, Wu ZH, Wang CN, Fang JH, Zhu L (2011). Synthesis and evaluation of aroylthiourea derivatives of 4-beta-amino-4’-O-demethyl-4-desoxypodophyllotoxin as novel topoisomerase II inhibitors. Eur. J. Med. Chem..

[17] Kumar NP, Sharma P, Reddy TS, Nekkanti S, Shankaraiah N, Lalita G (2017). Synthesis of 2,3,6,7-tetramethoxyphenanthren-9-amine: an efficient precursor to access new 4-aza-2,3-dihydropyridophenanthrenes as apoptosis inducing agents. Eur. J. Med. Chem..

[18] Kamal A, Kumar BA, Suresh P, Juvekar A, Zingde S (2011). Synthesis of 4beta-carbamoyl epipodophyllotoxins as potential antitumour agents. Bioorg. Med. Chem..

[19] Li WQ, Wang XL, Qian K, Liu YQ, Wang CY, Yang L (2013). Design, synthesis and potent cytotoxic activity of novel podophyllotoxin derivatives. Bioorg. Med. Chem..

[20] Sun WX, Ji YJ, Wan Y, Han HW, Lin HY, Lu GH (2017). Design and synthesis of piperazine acetate podophyllotoxin ester derivatives targeting tubulin depolymerization as new anticancer agents. Bioorg. Med. Chem. Lett..

[21] Tang ZB, Chen YZ, Zhao J, Guan XW, Bo YX, Chen SW (2016). Conjugates of podophyllotoxin and norcantharidin as dual inhibitors of topoisomerase and protein phosphatase 2A. Eur. J. Med. Chem..

[22] Zhang L, Chen F, Zhang Z, Chen Y, Lin Y, Wang J (2016). Design, synthesis and evaluation of the multidrug resistance-reversing activity of pyridine acid esters of podophyllotoxin in human leukemia cells. Bioorg. Med. Chem. Lett..

[23] Kumar A, Kumar V, Alegria AE, Malhotra SV (2011). Synthetic and application perspectives of azapodophyllotoxins: alternative scaffolds of podophyllotoxin. Curr. Med. Chem..

[24] Vander Heiden MG, Cantley LC, Thompson CB (2009). Understanding the Warburg effect: the metabolic requirements of cell proliferation. Science.

[25] Hanahan D, Weinberg RA (2011). Hallmarks of cancer: the next generation. Cell.

[26] Boyle P, Boniol M, Koechlin A, Robertson C, Valentini F, Coppens K (2012). Diabetes and breast cancer risk: a meta-analysis. Br. J. Cancer.

[27] Hardie DG, Carling D, Gamblin SJ (2011). AMP-activated protein kinase: also regulated by ADP?. Trends Biochem. Sci..

[28] Hardie DG (2013). AMPK: a target for drugs and natural products with effects on both diabetes and cancer. Diabetes.

[29] Mattison JA, Colman RJ, Beasley TM, Allison DB, Kemnitz JW, Roth GS (2017). Caloric restriction improves health and survival of rhesus monkeys. Nat. Commun..

[30] Zadra G, Photopoulos C, Tyekucheva S, Heidari P, Weng QP, Fedele G (2014). A novel direct activator of AMPK inhibits prostate cancer growth by blocking lipogenesis. EMBO Mol. Med..

[31] Viollet B, Horman S, Leclerc J, Lantier L, Foretz M, Billaud M (2010). AMPK inhibition in health and disease. Crit. Rev. Biochem Mol. Biol..

[32] Kovacic S, Soltys CL, Barr AJ, Shiojima I, Walsh K, Dyck JR (2003). Akt activity negatively regulates phosphorylation of AMP-activated protein kinase in the heart. J. Biol. Chem..

[33] Pommier Y, Leo E, Zhang H, Marchand C (2010). DNA topoisomerases and their poisoning by anticancer and antibacterial drugs. Chem. Biol..

[34] Li S, Lavagnino Z, Lemacon D, Kong L, Ustione A, Ng X (2019). Ca(2+)-stimulated AMPK-dependent phosphorylation of Exo1 protects stressed replication forks from aberrant resection. Mol. Cell.

[35] Shen Y, Sherman JW, Chen X, Wang R (2018). Phosphorylation of CDC25C by AMP-activated protein kinase mediates a metabolic checkpoint during cell-cycle G2/M-phase transition. J. Biol. Chem..

[36] Yuan L, Wang H, Liu Q, Wang Z, Zhang M, Zhao Y (2018). Etoposide-induced protein 2.4 functions as a regulator of the calcium ATPase and protects pancreatic beta-cell survival. J. Biol. Chem..

