# Supplementary material for: Novel Aza-podophyllotoxin derivative induces oxidative phosphorylation and cell death via AMPK activation in triple-negative breast cancer
Source: Br J Cancer. 2020 Nov 3;124(3):604–15. doi: 10.1038/s41416-020-01137-4 (PMC7851402; doi:10.1038/s41416-020-01137-4)
Supplement: Supplementary file 1 — Supporting material [file 41416_2020_1137_MOESM1_ESM.docx]

**Novel Aza-podophyllotoxin Derivative Induces Oxidative Phosphorylation and Cell Death *via* AMPK Activation in Triple Negative Breast Cancer**

Dhanir Tailor^1#^, Catherine C. Going^2#^, Angel Resendez^1^, Vineet Kumar^1^, Dhanya K. Nambiar^1^, Yang Li^1^, Arpit Dheeraj^1^, Edward Lewis LaGory^1^, Ali Ghoochani^2^, Alisha M. Birk^2^, Tanya Stoyanova^2, 5^, Jiangbin Ye^1^, Amato J. Giaccia^1, 5^, Quynh-Thu Le^1, 5^, Rana P. Singh^3^, George W. Sledge^4, 5^, Sharon J. Pitteri^2,5^*, Sanjay V. Malhotra^1,2,6*^

^1^Department of Radiation Oncology, Stanford University School of Medicine, Palo Alto, California 94304

^2^Department of Radiology, Canary Center at Stanford for Cancer Early Detection, Stanford University School of Medicine, Palo Alto, California 94304

^3^School of Life Sciences, Jawaharlal Nehru University, New Delhi, India 110067

^4^Department of Medicine, Stanford University School of Medicine, Palo Alto, California 94304

^5^Stanford Cancer Institute, Stanford University School of Medicine, Stanford, California 94305

^6^Department of Cell, Development and Cancer Biology, Knight Cancer Institute, Oregon Health & Science University, Portland Oregon 97201

Supporting Information

1. Synthesis of SU212 2
2. Materials and Methods 3
3. Supplementary figure S1-S3 7
4. References 12

**Synthesis of SU212 and drug preparation**

An aza-podophyllotoxin derivative named SU212 (Figure 1A) was synthesized and characterized as described previously ^37^. An aza-podophyllotoxin derivative named SU212 (Figure 1A) was synthesized and characterized as described previously ^37^. Briefly, a solution of amino alcohol component (2-((2,3-dihydro-1H-inden-5-yl)amino) ethanol, 0.177 g, 1 mmol), 3,4,5-trimethoxybenzaldehyde (0.235 g, 1.2 mmol), L-proline (0.011 g, 0.1 mmol, 10 mol %), and tetronic acid (0.120 g, 1.2 mmol) was prepared in anhydrous ethanol (4 mL) and the reaction mixture was refluxed for 3-4 h. Upon consumption of the amino alcohol component and the appearance of a fluorescent spot via thin layer chromatography (9:1 of 50% EtOAc/Hex:MeCN), a slurry of silica gel was prepared and purified by flash chromatography to yield the SU212 compound (0.230 g, 47%).

SU212. 0.230 g, 47% yield. 1H NMR (400 MHz, DMSO-d6) δ 7.06 (d, J = 17.5 Hz, 2H), 6.51 (s, 2H), 5.15 (d, J = 15.6 Hz, 1H), 5.05 (dd, J = 15.6, 1.1 Hz, 1H), 4.93 (dd, J = 5.5, 4.5 Hz, 1H), 4.83 (s, 1H), 4.00 – 3.89 (m, 1H), 3.70 (s, 0H), 3.67 (s, 6H), 3.67 – 3.57 (m, 1H), 3.58 (d, J = 1.7 Hz, 0H), 3.56 (s, 3H), 2.80 (t, J = 7.5 Hz, 2H), 2.70 (hept, J = 7.8, 7.3 Hz, 2H), 2.03 – 1.85 (m, 2H). 13C NMR (101 MHz, DMSO-d6) δ 172.74, 161.29, 153.19, 143.79, 139.30, 136.28, 134.93, 126.81, 125.23, 110.41, 104.96, 95.74, 66.21, 60.25, 58.30, 56.21, 48.16, 32.80, 32.00, 25.66. ESI-MS m/z calculated for C25H27NO6 [M+H] +: 438.180, found 438.1.

Aza-podophyllotoxin derivative stock solutions were made by dissolving in dimethyl sulfoxide (DMSO) to 2 mM concentration.

**Materials and Methods**

**Cell cycle phase distribution analysis**

30,000 cells/well were plated in a 12-well plate (Corning, USA). After 24 h the media was replaced with fresh media containing SU212 at 0.1, 0.25, or 0.5 µM for specified time periods (12 and 24 hours). Cells were collected and fixed in 70% ethanol. The fixed cells were stained using PI cocktail (80 μg/mL RNase A and 50 μg/mL propidium iodide in a saponin-EDTA solution in PBS) overnight at 4 °C and analyzed using a Guava easyCyte Flow Cytometer (MilliporeSigma, Burlington, MA). The percentage of cells in different phases of the cell cycle was calculated for control (DMSO) and SU212 treated samples after gating to remove debris and aggregates using FlowJo software.

**Western blot analysis**

MDA-MB-231 and MDA-MB-468 cells were seeded in 10 cm culture dishes (Corning) and incubated at 37 °C until the cells reached 60-70% confluence. The cells were then treated with SU212 at respective concentrations in fresh media for respective times. Whole cell lysates were prepared using M-PER™ Mammalian Protein Extraction Reagent containing Halt^TM^ Protease and Phosphatase Inhibitor Cocktail (Thermo Fisher Scientific, Waltham, MA). An equal amount of proteins were resolved on 10 or 12.5% or 4-12% SDS polyacrylamide gel electrophoresis and transferred onto a PVDF membrane (Bio-Rad, Hercules, CA). Blocked membranes were incubated with the respective primary antibody overnight at 4 °C, followed by HRP-conjugated secondary antibody incubation. The immunoreactive bands were visualized using an enhanced chemiluminescence ECL system (Immobilon Crescendo Western HRP substrate, MilliporeSigma, Burlington, MA) on an IVIS Lumina Imaging System (Perkin Elmer, Waltham, MA) and quantified using ImageJ (v1.8.0, NIH). Stripped blots were re-probed with an anti-β-actin antibody to normalize for differences in protein loading. The following antibodies were used for these studies: AMPKα (1:1000, Cat. # 5832S), pAMPKα (Thr172) (1:500, Cat. #2535S), Bcl-2 (1:1000 dilution, Cat. #2876S), Cyclin-B1 (1:1000 dilution, Cat. #12231T), CDC2 (1:2000 dilution, Cat. #9116T), p-Histone H3 (Ser10) (1:1000 dilution, Cat. #3377T), Bax (1:1000 dilution, Cat. #5023S), PARP (1:1000 dilution, Cat. #9542S), c-Caspase 3 (1:1000 dilution, Cat. #9664S), p-mTOR (Ser2448) (1:1000 dilution, Cat. #5536T), Beclin-1 (1:1000 dilution, Cat. #3495T), LC3 A/B (1:1000 dilution, Cat. # 12741T), Phospho-Acetyl-CoA Carboxylase (Ser79) (pACC) (1:1000 dilution, Cat. #11818T), Acetyl-CoA Carboxylase (ACC) (1:1000 dilution, Cat. #3676T) purchased from Cell Signaling Technology (Danvers, MA), β-actin (1:10000 dilution, Cat. #NB600-501) purchased from Novus Biologicals (Littleton, CO), and mTOR (1:1000 dilution, Cat. #659201) purchased from BioLegend, Inc (San Diego, CA).

**Oil Red O staining**

1x10^5^ cells were plated per well in 6 well plates and allowed to attach for 24 h. Cells were treated with 0.25 or 0.5 μM SU212 for 12 h. At the end of treatment, cells were fixed with 10% neutral buffered formalin. Cells were washed with deionized (DI) water and incubated with 60% isopropanol for 5 minutes followed by ORO staining (0.5% ORO in 100% isopropanol, diluted with DI water in the ratio of 3:2) (Electron Microscopy Sciences, PA, USA) for 30 minutes. The stain was removed, and cells were washed with DI water to get a clear background. Images for each well were captured using bright field microscopy at 20X magnification. The stain was eluted by 100% isopropanol and absorbance was measured at 500 nm to quantify lipid content.

**Proteomic Analysis**

For proteomics experiments, TNBC cell lines MDA-MB-231 and MDA-MB-468 as well as normal breast cell lines MCF10A and MCF12A were grown to 80% confluence in 10 cm plates and then treated with either 0.5 µM SU212 diluted from a 20 mM stock solution in DMSO or with DMSO vehicle control. A concentration of 0.5 µM SU212 was chosen because it is the lowest concentration at which a significant difference in cell viability between TNBC and normal cells was observed in the viability assay, and cells were harvested after 12 hours treatment to minimize apoptosis.

Cells were lysed, and proteins were precipitated, digested into peptides, and labeled with Tandem Mass Tag (TMT) six-plex reagents (Thermo Fisher Scientific) according to the manufacturer's protocol. Samples were analyzed by liquid chromatography-tandem mass spectrometry (LC-MS/MS) on a Dionex Ultimate 3000 LC (Thermo Fisher Scientific) coupled to an LTQ-Orbitrap Elite (Thermo Fisher Scientific) mass spectrometer using high energy collision-induced dissociation (HCD). Protein identification and quantification was performed using MaxQuant ^38^ and Perseus ^39^. Pathway enrichment was performed using Gene Set Enrichment Analysis ^40^, with proteins ranked according to log_2_(fold change upon treatment).

**C13-Glucose tracer assay**

C3 glucose tracing was measured by following protocol previously described by Tarangelo et al. 2018 ^41^. Briefly, 2 x 10^5^ cells were plated in 60 mm dishes. At 60-70% confluence, cells were washed with PBS and media was replaced with fresh media containing C13-glucose and dialyzed FBS. Cells were incubated for respective time points (1 and 6 h) and lysed using ice-cold 80% acetonitrile. Cell debris was removed via centrifugation at 12000 rpm for 10min and supernatant was collected in glass vials. Each sample was analyzed using LC-MS/MS to measure the metabolic conversion of C13 -glucose. Total protein in the pellet was measured using a BCA protein assay and used to normalize each sample.


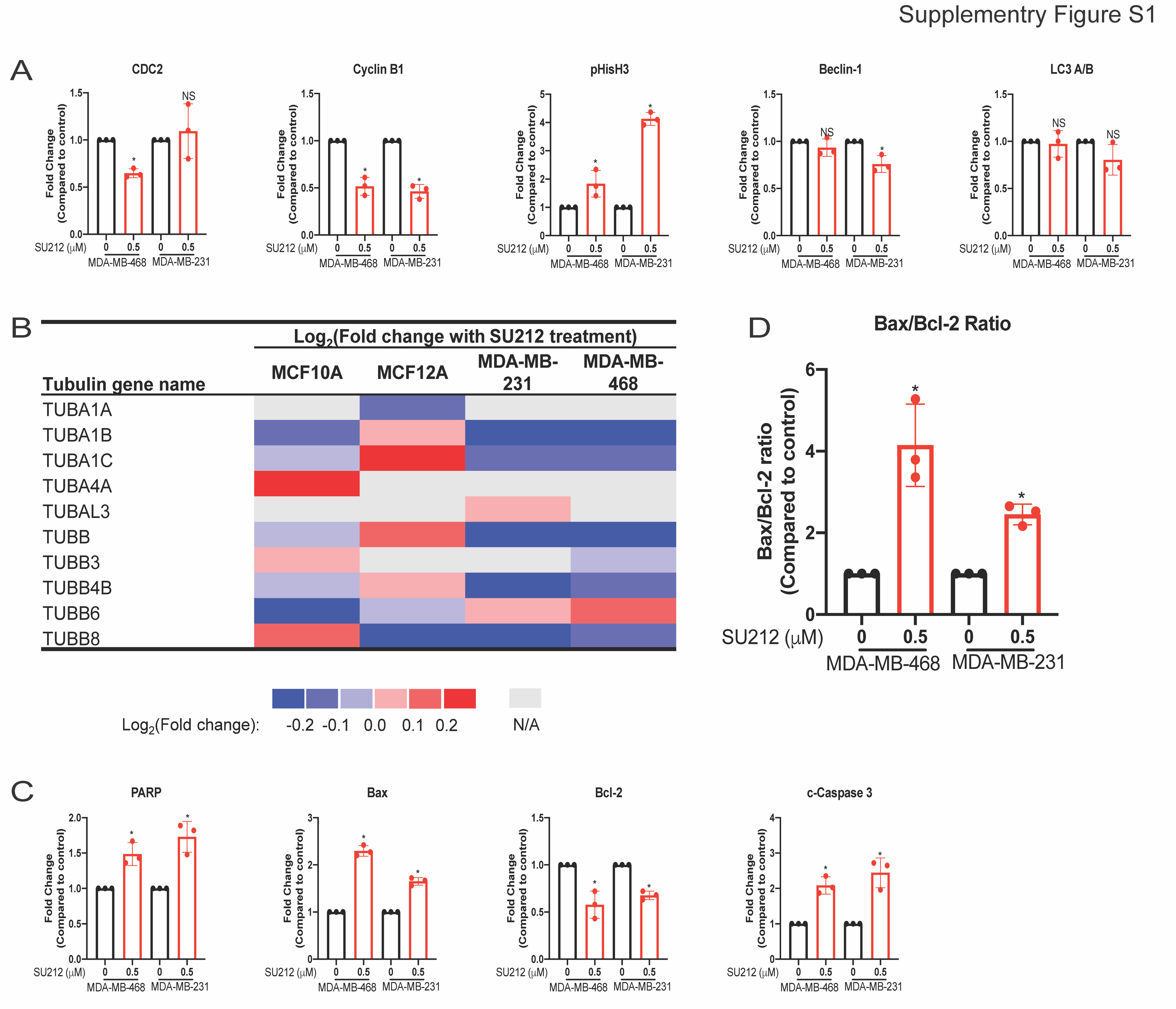


**Supplementary figure S1:** **A**, TNBC cells were treated with SU212 for 6 h, and total cell lysates were prepared as described in the Methods section. SDS-PAGE and western blot analysis were performed for cell cycle and autophagic markers. Membranes were stripped and re-probed with anti-beta-actin antibody to ensure equal protein loading. Band intensity was quantified using ImageJ software and normalized compared to control. Quantitative data was plotted from three independent biological replicates. **B**, GSEA was performed on the proteomics results to determine the enrichment of KEGG pathways upon treatment of MCF10A and MCF12A or TNBC MDA-MB-231 and MDA-MB-468 cell lines with SU212 (12h, 0.5 μM). Enrichment in the gap junction pathway (p-values of 0.03 and 0.16 for MDA-MB-231 and MDA-MB-468, respectively) was observed in proteins that decrease in abundance upon treatment with SU212 for TNBC cell lines mainly due to decreases in the abundance of tubulins. **C**, TNBC cells were treated with SU212 for 12 h and total cell lysates were prepared as described in the Methods section. SDS-PAGE and western blot analysis were performed for apoptotic cell death markers and Quantitative data was plotted from three independent biological replicates. **D**, Bax/Bcl-2 ratio was calculated from protein quantification data and plotted cumulatively from three independent biological replicates. Data shown was mean ± SD of three independent plates; each sample was counted in duplicate. Data was analyzed using one-way ANOVA Dunnett's test. * indicates p < 0.05, significantly different compared with corresponding control.


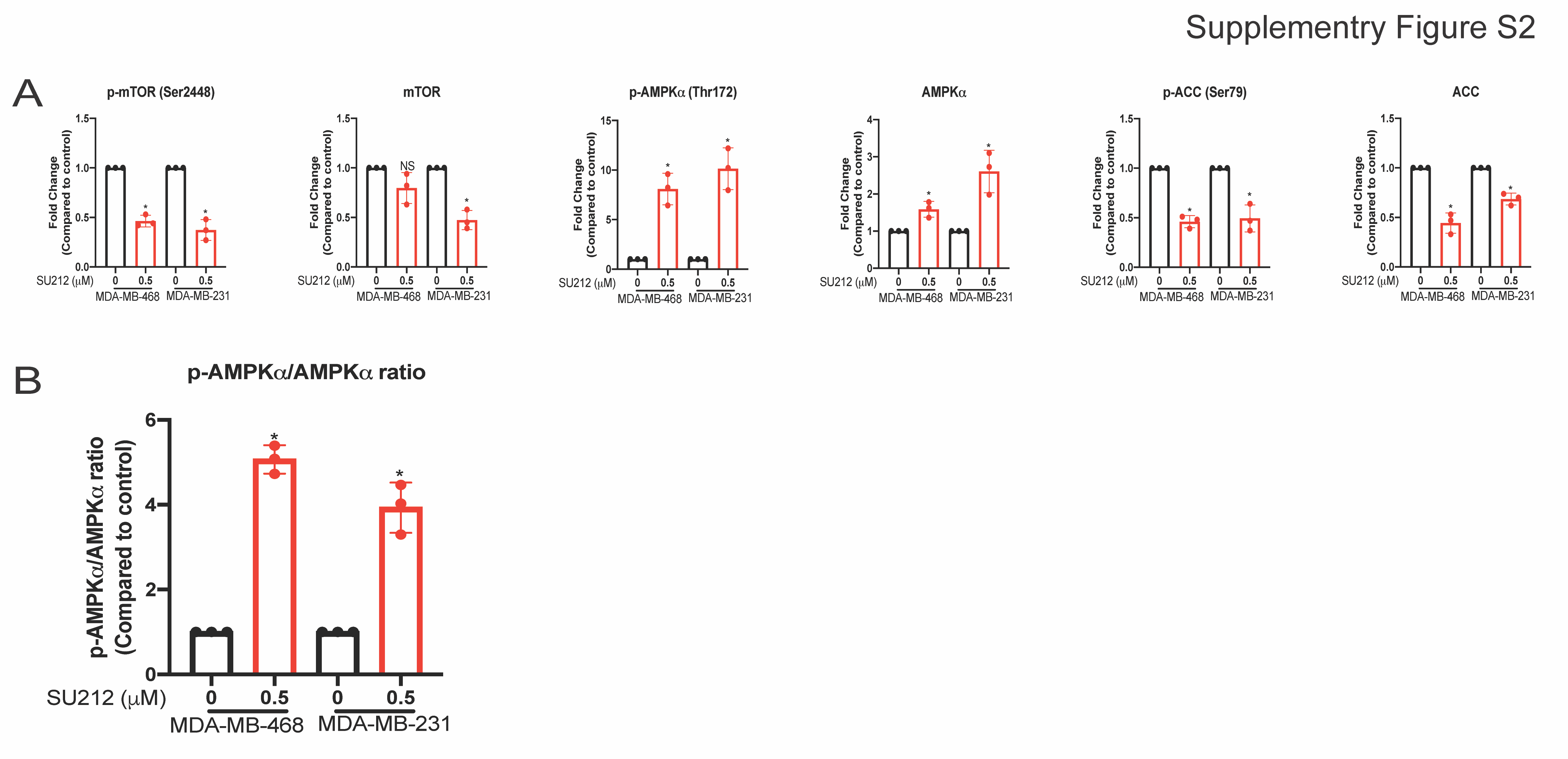


**Supplementary figure S2:** TNBC cells were treated with SU212 for 6 h and total cell lysates were prepared as described in the Methods section. SDS-PAGE and western blot analysis were performed for AMPK pathway associated proteins. Membranes were stripped and re-probed with anti-beta-actin antibody to ensure equal protein loading. Band intensity was quantified using ImageJ software and normalized compared to control. **A**, Quantitative data of protein quantification was plotted from three independent biological replicates. **B**, p-AMPKα/AMPKα ratio was calculated from protein quantification data. Data shown was mean ± SD of three independent plates; each sample was counted in duplicate. Data was analyzed using one-way ANOVA Dunnett's test. * indicates p < 0.05, significantly different compared with corresponding control.


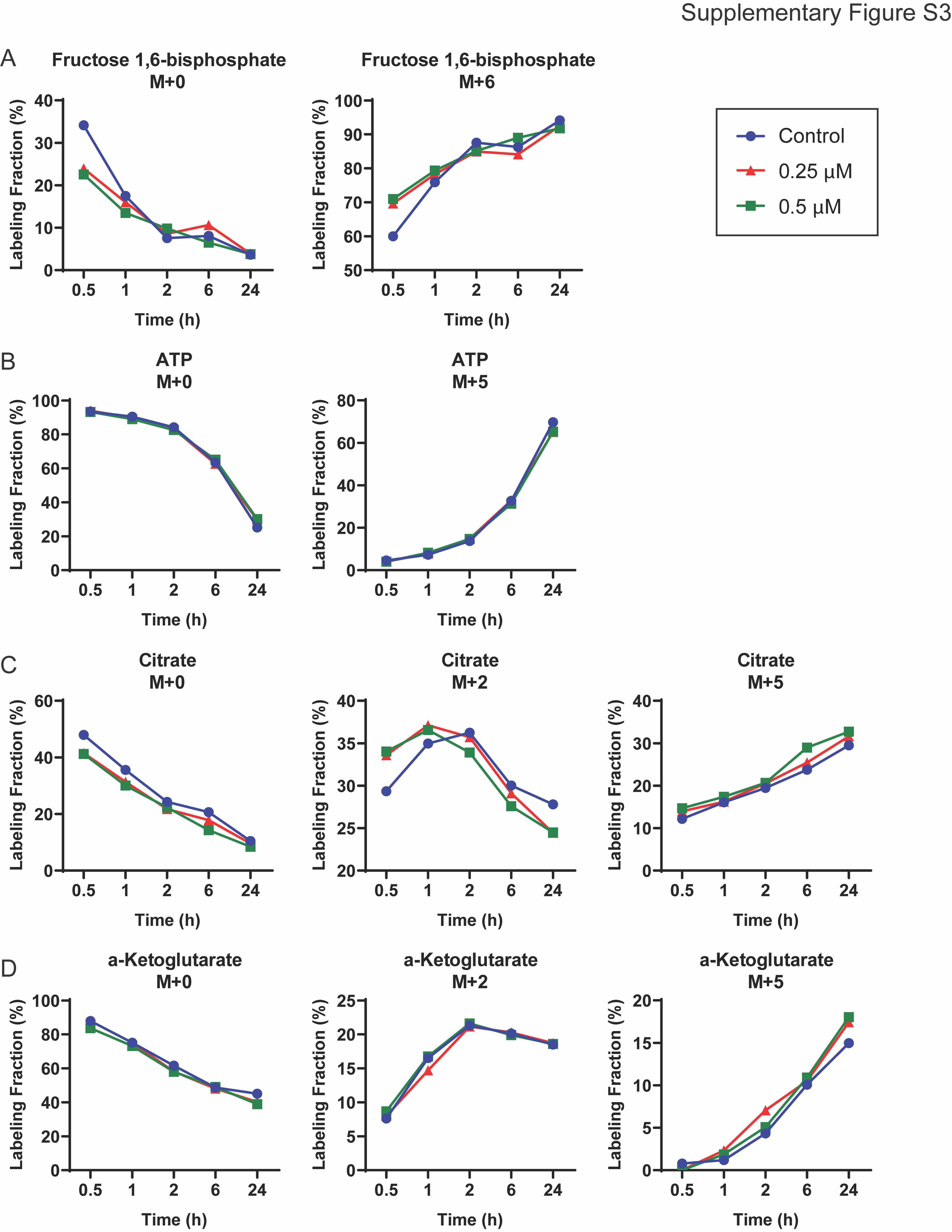


**Supplementary figure S3:** **A-D**, C13-Glucose tracking assay. Cells were fed with C13-glucose-containing media, and cells were lysed and analyzed using LC-MS/MS for C13 tracing. Each graph shows the cellular level of each component at each time point in vehicle and SU212 (0.25 and 0.5 µM) treated cells. **A**, Fructose 1-6 bisphosphate. **B**, ATP. **C**, Citrate. **D**, α-ketoglutarate.

**Reference:**

37. Kim YS, Kumar V, Lee S, Iwai A, Neckers L, Malhotra SV *et al.* Methoxychalcone Inhibitors of Androgen Receptor Translocation and Function. *Bioorganic & medicinal chemistry letters* 2012; **22**(5): 2105-2109; doi 10.1016/j.bmcl.2011.12.141.

38. Cox J, Mann M. MaxQuant enables high peptide identification rates, individualized p.p.b.-range mass accuracies and proteome-wide protein quantification. *Nat Biotechnol* 2008; **26**(12): 1367-1372; e-pub ahead of print 2008/11/26; doi 10.1038/nbt.1511.

39. Tyanova S, Temu T, Sinitcyn P, Carlson A, Hein MY, Geiger T *et al.* The Perseus computational platform for comprehensive analysis of (prote)omics data. *Nat Methods* 2016; **13**(9): 731-740; e-pub ahead of print 2016/06/28; doi 10.1038/nmeth.3901.

40. Subramanian A, Tamayo P, Mootha VK, Mukherjee S, Ebert BL, Gillette MA *et al.* Gene set enrichment analysis: a knowledge-based approach for interpreting genome-wide expression profiles. *Proc Natl Acad Sci U S A* 2005; **102**(43): 15545-15550; e-pub ahead of print 2005/10/04; doi 10.1073/pnas.0506580102.

41. Tarangelo A, Magtanong L, Bieging-Rolett KT, Li Y, Ye J, Attardi LD *et al.* p53 Suppresses Metabolic Stress-Induced Ferroptosis in Cancer Cells. *Cell Rep* 2018; **22**(3): 569-575; e-pub ahead of print 2018/01/19; doi 10.1016/j.celrep.2017.12.077.
